# The Impact of Internet Usage and Knowledge-Intensive Activities on Households’ Healthcare Expenditures

**DOI:** 10.3390/ijerph17124470

**Published:** 2020-06-22

**Authors:** Marco Benvenuto, Alexandru Avram, Francesco Vincenzo Sambati, Marioara Avram, Carmine Viola

**Affiliations:** 1 Department of Economics, University of Salento, 73100 Lecce, Italy; francesco.sambati@unisalento.it (F.V.S.); carmine.viola@unisalento.it (C.V.); 2 Department of Finance, Faculty of Economics and Business Administration, West University of Timisoara, 300115 Timisoara, Romania; alexandru.avram@e-uvt.ro; 3 Department of Economics, Accounting and International Affairs, University of Craiova, 13, A. I. Cuza Street, 200585 Craiova, Romania; marioaraavram@yahoo.com

**Keywords:** internet usage, knowledge intensive activities, healthcare expenditure, decision making, vector autoregressive model

## Abstract

This paper examines the impact of the internet usage and knowledge intensive activities on households’ healthcare expenditures Similarly, the paper aims to recognize and understand, from a value-creation perspective, the correlation between: internet access of households (IA), individuals frequently using the internet (IU), individuals searching on internet for health-related information (HI), payments made by households for healthcare (PHH), expressed as euro per inhabitant and employment in knowledge-intensive activities (KIA). The approach utilized in the present study consists of two steps. First, a theoretical framework was conducted to determine the existing relationship between major variables. Next, the Vector Autoregressive (VAR) approach was applied in a case study at European level to prove the three hypothesis we consider. By analyzing the connection between the major variables, a positive and long- lasting impulse response function was revealed, followed by an ascending trend. This suggests that a self-multiplying effect is being generated; and it reasonable to assume that the more individuals use the Internet, the more electronic acquisitions occur. We can thus reasonably conclude that the improvement of the internet usage and knowledge intensive activities on households’ healthcare expenditures process is strongly dependent on people’s capability. Improving IU and KIA is the new reading key in the decision-making process in health system approach.

## 1. Introduction

Internet usage (IU) plays a crucial role in Supply Chain Management (SCM), and the robust published literature confirms its centrality in transforming today’s health management system. However, there is a significant debate on impact of internet usage and knowledge intensive (KIA) activities in SCM. IU offers vast prospects for developing a collaborative system model that can create synergies, both inside and outside the healthcare scenarios. The opportunities offered by IU are indeed really promising, especially for improving management decisions, implementing robust risk management and operational efficiency, enhancing KIA innovation capabilities, and creating a better overall customer experience.

This approach is developing patchily, with social, technological, and human health consequences that need to be considered in order to provide real long-term advantages for households’ healthcare, both in terms of health efficiency and also from a cost reduction perspective. There are several obstacles to using IU appropriately, including security, privacy, ethics, and governance health expenditure issues.

The paper aims to measure the impact of IU and KI activities on households’ healthcare expenditures, to show how these critical aspects can represent a useful tool in the decision-making process of SCM and to emphasize the existing correlation between them. Due to the complexity of the subject matter, we followed a tiered approach, dividing the research work into two steps. First, we identified through a theoretical framework a previously hidden relationship among Internet Usage, Health Information and Knowledge-Intensive Activities. This first step allowed us to identify the variables needed to recognize the impact more concretely, and to design a case study (step two) that could contextualize the revealed relationship. Accordingly, a Vector Autoregressive (VAR) model was applied to conduct empirical research on European context.

Based on the above-mentioned approach, the article is organized as follows: the introduction presents the challenges and general goals of the present research, while Section 2 offers working definitions of internet usage and knowledge intensive activities on households in healthcare system as a preliminary research step. Next, Section 3 presents the results of a longitudinal empirical study of European State, using a VAR model for its implementation. Section 4 presents the study’s conclusions and discussion, including research implications for the practical use of the outputs and future research possibilities on the paper’s topic.

## 2. Context Description

### 2.1. Health Population

Nowadays we are facing the demographic change. In Europe it is estimated that over one third of the population in 2025 will be more than 60 years old, in addition the older population aged between 65 and 79 has increased significantly since 2000 [1] and this trend will be constant until 2050 [2]. The population explosion we are observing today originates from the post-conflict Baby Boom and will begin to mitigate its effects only starting from 2030. Life expectancy in Europe has significantly improved in the period between 1960 and 2002 due to a significant reduction in mortality in all population groups [3]. For example, in countries such as Iceland, Switzerland, Sweden the life expectancy at birth is found between 80.2 and 80.6 years. Healthy life expectancy considers the life expectancy including the time spent in poor health, it corresponds to the number of healthy years that a person who has reached the age of 60 expects to live on the basis of current mortality rates and bad health conditions [4].

In European countries the state of health of elderly people points out the prevalence of physical problems. The most commonly reported chronic conditions include arthritis diabetes and heart disease, while the main causes of death concern cardio-circulatory diseases and malignant neoplasms [5].

According to the World Health Organization, we can distinguish diseases in two main categories: infectious and non-communicable. Those infectious are generally caused by pathogenic microorganisms and spread directly or indirectly between people, while non-communicable ones (NCDs) also known as chronic diseases and tend to accompany an individual throughout his life.

Most NCDs are associated with older population groups but the reality shows that over 15 million deaths among the population aged between 30 and 69 years are attributed to non-communicable diseases: this makes us understand how the entire population is at risk of contracting at least one non-communicable pathology. Even if NCDs show a global reduction they still kill over 41 million people worldwide and they are the cause of death of 15 million people between the ages of 30 and 69 years. World Health Organization suggests the best way to control non-communicable diseases is to focus on reducing the risk factors associated with them, boosting interventions focused on prevention and control [6]; in fact, the European Community through its website is promoting actions aimed at spreading health behaviors for health promotion and disease prevention among the population [7].

In Europe, public health spending is growing fast among governments. In 2015 [8] it averaged around 7.8% of the gross domestic product with over 70% of health expenses covered by the public sector (in over two thirds of the member countries). According to recent reports, premature deaths were recorded in 2013 due to the main non-communicable diseases, causing a cost to European Community economies of 0.8% gross domestic product. Due to the aging of the population, the increase in cases of chronic diseases and due to the spread of new diagnostic therapeutic technologies [9,10] an increase in the share of gross domestic product in the health sector is expected in the near future [8]. This consideration is even more true in those countries characterized by a medium-high income, in fact non-communicable diseases absorb a significant share of health costs [11]. it is estimated that in 2015 cardiovascular diseases cost around 111 billion €, while those related to cardiac and ischemic heart disease are respectively 19 billion € and 20 billion € [12].

According to OECD most healthcare costs are attributed to the largest non-communicable diseases [13], and there is a per capita health expenditure among the very elderly population with a high difference over 85 and the population aged between 55 and 59 years.

### 2.2. The Internet, Health Information and Knowledge-Intensive Activities

One of the main tools currently used to disseminate best practices regarding the lifestyles and habits of the population as well as information necessary to maintain good health status is undoubtedly the Internet [14].

The internet was defined as a global connection tool for individual networks run by governments, industry, universities and private actors. Originally conceived to connect few actors, mostly institutional, since 1994 it has expanded to serve billions of users for a multitude of purposes anywhere in the world, this connection tool has turned into a formidable platform that has upset the way we do business and the more generically we communicate, guaranteeing a globalized dimension to the whole world. Currently it has become a universal source of information for the entire world population, if in case you are at home, at work, at school or in your spare time.

Another element that characterizes the internet has been its evolution which has not found any precedent in the past. This element is characterized by two applications, the social web and mobile technology. They have generated a significant break with the traditional use of the internet. One of the most significant examples of social web is Facebook, launched in 2004 grew into the global network reaching 4422 million of active users. On the other hand, mobile technology has enabled greater internet coverage, allowing anyone to be connected anywhere [15].

From the democratization of the Internet, ITU, OECD and Eurostat, together with other important world organizations, they have discussed and developed a list of indicators to measure the level of diffusion and the impact of information and communication technologies (ICT). Since 2005, over 50 indicators covering different areas have been developed, such as: ICT infrastructure and access; access and use of ICT by households and individuals; use of ICT by businesses; the ICT sector; trade in ICT goods; ICT in education; and e-government [16].

From the last REPORT of ITU [17] it emerged that starting from 2005 the “Mobile-cellular telephone subscriptions” raised from about 40 percent in 2005 to 107 subscriptions per 100 inhabitants in 2018. The most significant growth was recorded for “Active mobile-broadband subscriptions”, which increased from 5% in 2007 to 69.3% in 2018. Another interesting element is related to the individuals that use the internet, in fact starting from 2005 until 2018 the percentage of population that uses the Internet has more than doubled starting from 18% up to 51%. While on the one hand the International Telecommunication Union reports a progressive reduction in the subscription of fixed telephony contracts on the other, it highlights how broadband internet access is progressively growing globally, passing from 3% in 2005 to over 14% in 2018. These data are more evident if we focus on the 2015–2017 three-year period: in the European area, in America and in Asia it is evident how the broadband connection progressively replacing the old connections (2G/3G). This phenomenon is more mitigated in the areas of Africa and the Arab states, highlighting the problem of inequality in internet access. With the Internet and his diffusion came a problem, spread both globally and locally: the so-called “Digital Divide” [18]. In some studies, it emerged that disparities in internet usage and internet access could emerge between countries and within counties [18,19]. The Digital Divide, according to Norris is related to three main key elements:

(1) Infrastructure [20] and hardware [21] adopted;
(2) Socio-economic dimension [22,23];
(3) Users’ ability to use internet related devices [19,24,25,26]

A relevant activity online, among adult and older people, it is to google health information: “…any information which is related to the practice of medicine and health-care…” and “…information which can aid in the prevention, detection, and treatment of disease…’’ [27]. Internet transformed the way to find information, it provided the fastest, richest and up-to-date information to clinicians and patients. He also highlighted that the Internet penetration, among American homes, has revealed a problem with the skills needed to find, evaluate and understand information (especially health information). Contextually, the increasing age (older people) and the incidence of chronic diseases increased the likelihood to seek out health related news and information. Some scholars stated that platforms, that facilitate exchange information in the internet, are responsible for using cell phone to search health or medical information [28]. Internet usage and health information seeking is also related to the age of “user”, in fact a research of 2009 showed the seniors people are most likely to search online health information to maintain their health condition or to manage their chronic disease [29], while others researchers [30] reported that “members of medically underserved groups are less likely to use the Internet for health information”, that internet access is preparatory to online health information search and that Internet use (time, frequency, or activities) is related to the frequency of online health information search.

According to the third key element of the Digital Divide, the numbers of employees in Knowledge-intensive service and activities (also known as KISA or KIA), could be considered as a proxy. KISA (or KIA) are knowledge-intensive services that include—according to OCSE definition [31]: High-tech knowledge-intensive services, Knowledge-intensive market services, Knowledge-intensive financial services and Other knowledge-intensive services (such as: publishing activities, education, human health activities, etc.). “In 2018, 35.3 million people were employed in the manufacturing sector in the EU-28, a figure which represented 15.4% of total employment. Of these, 2.4 million were employed in high-tech manufacturing, corresponding to 1.1% of total employment. Almost three times as many were employed in high-tech knowledge intensive services, which accounted for 3.0% of total employment” [32]. In the 2003, Madsen et al., analyzed the importance of human capital in the knowledge-intensive entrepreneurship [33]. Authors observed that, in knowledge-intensive sectors, entrepreneurs were generally high educated and, in BIOMED sector, “all entrepreneurs have a master’s degree, but more than 65 per cent have a PhD”. Domenech et al. [34], showed “Regions with large numbers of highly skilled workers have high rates of employment in knowledge-intensive activities” and that regions, with high portion of highly qualified workers, are able to give back public R&D expenditures in terms of effectiveness. In addition, studies show the positive externalities of keeping a high rate of health safety for the households [35]. Thus, in *caeteris paribus conditions* keeping a high rate of health safety for the human capital means having a high rate of occupational flexicurity [36] and thus more efficient financial decisions and better usage of financial capital orientated towards human resource management [37,38].

In conclusion, we can assert that exist three research field to deeper. They can be synthetized in this way:

(1) Internet access of households is influencing in a positive manner the internet usage and the Knowledge intensive activities.
(2) Internet usage is influencing in a positive manner both the search for healthcare information on the internet and also the knowledge intensive activities.
(3) Internet search for healthcare is affecting the spending of households on healthcare.

In the next section we applied an empirical methodology in order to explain this theoretical framework.

## 3. Materials and Methods

Our study is based on the fact that more and more individual are using the internet as a source of information for treatment for health issues. In order to assess the implications for this widespread phenomenon, we have chosen the following variables: internet access of households (IA), individuals frequently using the internet (IU), individuals searching on internet for health related information (HI), payments made by households for healthcare (PHH), expressed as euro per inhabitants and employment in knowledge-intensive activities (KI).

Using Eurostat data, our variables that were taken into consideration were:

- *Internet access of households (IA)*: the statistics is presenting the use within househoulds of Information and Communication Technologies at European level. Data for this collection are supplied directly from the surveys with no separate treatment, presenting the access to and use of ICTs by individuals and/or in households and the use of the Internet and other electronic networks for different purposes by individuals and/or in households;
- *Individuals frequently using the internet (IU)*: the statistics is presenting the access to and use of ICTs by individuals in order to measure the access to selected IC technologies, the use of computers, location, frequency of use, activities (data collected at individual level), the use of the Internet (data collected at individual level), internet commerce (data collected at individual level), e-skills;
- *Individuals searching on the internet for health-related information (HI)*: the statistics is presenting the percentage of individuals seeking Health-related information, such as: injury, disease, nutrition, improving health, etc. The data is collected taking into consideration the last three months before the survey.
- *Payments made by households for healthcare (PHH)*: the statistics is presenting household payment’ means a direct payment for healthcare goods and services from the household primary income or savings, where the payment is made by the user at the time of the purchase of goods or the use of the services. The statistics is expressed as euro per inhabitants.
- *Employment in knowledge-intensive activities (KI)*: the statistics is presenting employment in Knowledge Intensive Activities (KIA) identified based on a level of educated persons in sectors of economic activity. The data is covering EU Member States, EFTA and candidate countries are extracted and compiled from EU-Labour Force Survey for the population, using annual average data.

The VAR model used is an unrestricted panel VAR, since the variables are integrals of different orders. The Model proposed by us is original and observes the recommendations of Harvey [39], in 1990.

The VAR methodology is widely used in literature due to the large applicability, regardless of the field and topic. VAR models do not require in depth knowledge about the influences between dependent and independent variables but rather a list of variables which can be hypothesized to affect each other intertemporally.

Thus, because of the interdependencies between our variables, the impact between them being mutual, we have decided to use a VAR Panel model, one the most versatile research methodology available in our research inventory. Therefore, we taken 27 cross-sections to the European member states, excluding only Malta, due to the lack of data. The period taken into consideration spans across 10 years, from 2007, until 2016.

According to Canova and Cicarelli (2013) [40] panel VAR models are best at determining the explicit microstructure present in DSGE models and, as their VAR counterparts, attempt to capture the dynamic interdependencies present in the data using a minimal set of restrictions.

The data used were collected from Eurostat, using the latest data available, starting from the date of 2007, until 2016, making sure they were seasonally adjusted and complete for a using the panel methodology. In addition, the data were differentiated by first degree, and a logarithm was used in order to respond to the tests needed for the VAR methodology.

On the basis of the results of literature review we have formulated 3 major hypotheses, as follows:

**Hypothesis 1** **(H1).** *Internet access of households is influencing in a positive manner the internet usage and the Knowledge intensive activities. Thus, in our opinion, the higher the internet access, the higher the internet usage of households and the knowledge intensive activities.*

**Hypothesis 2** **(H2).** *Internet usage is influencing in a positive manner both the search for healthcare information on the internet and also the knowledge intensive activities. Thus, in our opinion, greater internet usage develops know how and insight in health-related issues.*

**Hypothesis 3** **(H3).** *Internet search for healthcare is having a negative influence on the spending of households on healthcare. So, we are stating that by searching information on health on the internet, the members of the households can make informed and thus better decisions and subsequently, increase the efficiency of the spending.*

The main reason we have chosen the VAR approach is that VAR is a natural tool for forecasting [41], focusing mainly on the impulse response functions and variance decomposition as the main tools for disentangling the VAR relations. We have analyzed the impulse response functions for describing the relationship between the variables chosen as follows:

(1) $$IA_{1,t}=\alpha_{2}+∆\overset{j}{\underset{j=1}{\sum }}\beta_{1,j}IA_{1,t-j}+∆\overset{j}{\underset{j=1}{\sum }}\delta_{1,j}IU_{1,t-j}+∆\overset{k}{\underset{k=1}{\sum }}\varepsilon_{1,j}HI_{1,t-j}+∆\overset{k}{\underset{k=1}{\sum }}\epsilon_{1,j}PHH_{1,t-j}+∆\overset{k}{\underset{k=1}{\sum }}\epsilon_{1,j}KI_{1,t-j}+u1_{t}$$

(2) $$IU_{1,t}=\alpha_{2}+∆\overset{j}{\underset{j=1}{\sum }}\beta_{1,j}IU_{1,t-j}+∆\overset{j}{\underset{j=1}{\sum }}\delta_{1,j}IA_{1,t-j}+∆\overset{k}{\underset{k=1}{\sum }}\varepsilon_{1,j}HI_{1,t-j}+∆\overset{k}{\underset{k=1}{\sum }}\epsilon_{1,j}PHH_{1,t-j}+∆\overset{k}{\underset{k=1}{\sum }}\epsilon_{1,j}KI_{1,t-j}+u1_{t}$$

(3) $$HI_{1,t}=\alpha_{2}+∆\overset{j}{\underset{j=1}{\sum }}\beta_{1,j}HI_{1,t-j}+∆\overset{j}{\underset{j=1}{\sum }}\delta_{1,j}IU_{1,t-j}+∆\overset{k}{\underset{k=1}{\sum }}\varepsilon_{1,j}IA_{1,t-j}+∆\overset{k}{\underset{k=1}{\sum }}\epsilon_{1,j}PHH_{1,t-j}+∆\overset{k}{\underset{k=1}{\sum }}\epsilon_{1,j}KI_{1,t-j}+u1_{t}$$

(4) $$PHH_{1,t}=\alpha_{2}+∆\overset{j}{\underset{j=1}{\sum }}\beta_{1,j}PHH_{1,t-j}+∆\overset{j}{\underset{j=1}{\sum }}\delta_{1,j}IU_{1,t-j}+∆\overset{k}{\underset{k=1}{\sum }}\varepsilon_{1,j}HI_{1,t-j}+∆\overset{k}{\underset{k=1}{\sum }}\epsilon_{1,j}IA_{1,t-j}+∆\overset{k}{\underset{k=1}{\sum }}\epsilon_{1,j}KI_{1,t-j}+u1_{t}$$

(5) $$KI_{1,t}=\alpha_{2}+∆\overset{j}{\underset{j=1}{\sum }}\beta_{1,j}KI_{1,t-j}+∆\overset{j}{\underset{j=1}{\sum }}\delta_{1,j}IU_{1,t-j}+∆\overset{k}{\underset{k=1}{\sum }}\varepsilon_{1,j}HI_{1,t-j}+∆\overset{k}{\underset{k=1}{\sum }}\epsilon_{1,j}IA_{1,t-j}+∆\overset{k}{\underset{k=1}{\sum }}\epsilon_{1,j}PHH_{1,t-j}+u1_{t}$$

where IA = internet access of households; IU = individuals frequently using the internet; HI = individuals searching on internet for health-related information; PHH = payments made by households for healthcare, expressed as euro per inhabitants and KI = employment in knowledge-intensive activities

After we have estimated the VAR, we have tested whether our model is satisfying all the necessary VAR tests. Thus, the VAR satisfies the stability condition as shown in Table 1.

Therefore, we have ensured the essential condition for VAR stability. From this point on, we can continue our first study. According to Table 2, the number of lags is chosen as 1. Thus, a change with a unit of the independent variable will have an effect on the dependent variable within the nest period of time, thus almost instant. We believe this lag is correct, as the consumer will formulate its own set of searches on the internet. However, the search for health-related information is not likely to come as soon as he/she will have access to the internet. In one period of time is very likely that every individual can and will search the internet for a health-related problem.

The VAR satisfies the autocorrelation condition, as shown in Table 3. This condition is respected by using the Portmanteau autocorrelation test, being an essential condition in validating the VAR methodology.

Also, one can see in Table 4 that the VAR model is respecting the Heteroskedasticity conditions and therefore we can proceed forward with our impulse response functions.

Following the verification of VAR stability conditions and the tests ensuring data accuracy, we performed the impulse functions to observe the impact of the of the variables selected and to confirm our hypothesis.

## 4. Results

Following the validation of the model thru the tests presented in Table 1, Table 2, Table 3 and Table 4, we have moved forward to perform the impulse response function specific to VAR. The number of lags chosen, as we can observe in Table 2, is one. Thus we will have effects of the dependent variable will be observable during the next period of time, thus the effects are rapid and consistent as we can observe in the impulse response functions presented below.

### 4.1. The Relationship between Internet Access and Internet Usage

**Figure 1:**
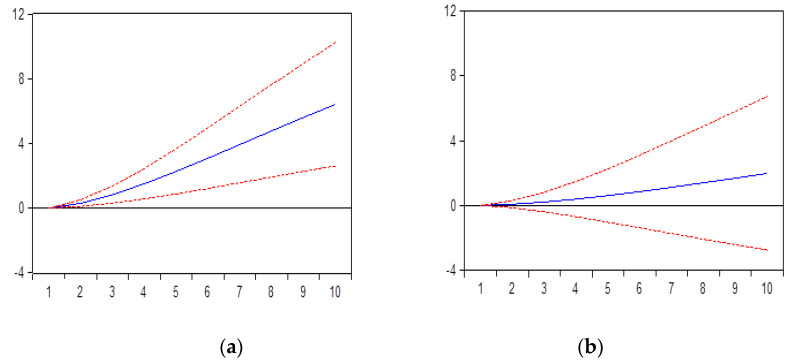
(**a**) Impact of HI to IU; (**b**) Impact of HI to KI. Impact of internet usage and employment in knowledge intensive activities on individuals searching for health-related information.

As we can observe from the Figure 1a,b, the internet access will influence in a positive manner the internet usage. It makes perfect sense that this relationship works in a mutual way, both variables are influencing each other. Therefore, the higher the access (IA), the higher the usage (IU). But also, the internet usage has a positive impact on internet health related issues. In addition, the higher the employment in knowledge-intensive activities (KI), the higher the number of individuals searching on internet for health-related information (HI). As we can observe, the impact is strong, long lasting for the entire period analyzed.

### 4.2. The Relationship between Internet Usage and Internet Search for Seeking Health Related Information

**Figure 2:**
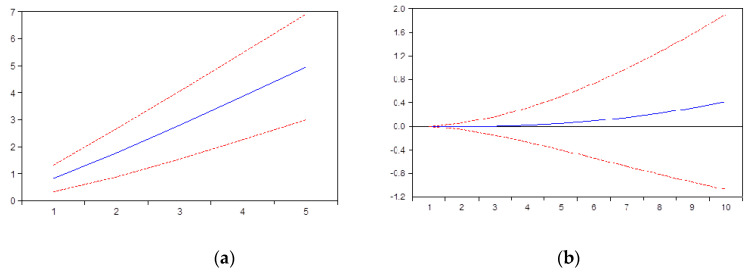
(**a**) Impact of Hi to IU; (**b**) Impact of Hi to PHH. Impact of internet usage and payments made by households for healthcare, expressed as euro per inhabitants.

Our second hypothesis is also confirmed by the impulse response function. Thus, as we can observe in Figure 2a there is a positive response of HI to IU. We can explain this in a very simple manner, that the higher the access to internet, the more information regarding health is expected to be searched. The impulse response function shows a very strong correlation, an upward trend and persistent effects. In addition, Figure 2b shows that HI also responds positive to PHH. In our opinion, the larger the payments made by households for health-related expenses, the higher the number of searches on internet.

### 4.3. The Relationship between Using Internet for Health-Related Information and Payments Made by Households for Healthcare Expenses

**Figure 3:**
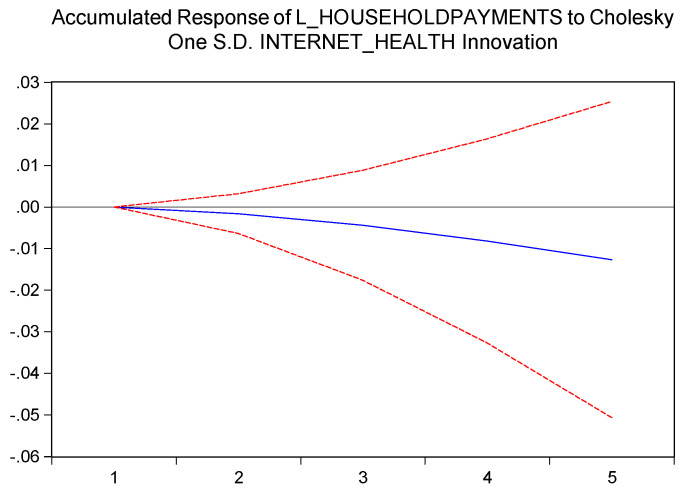
Impact of individuals searching on internet for health-related information on payments made by households for healthcare, expressed as euro per inhabitants.

Out third hypothesis is also confirmed, as we ca see in Figure 3. We can state that the more access to information the individuals are having, the lower the possibility of accessing healthcare services in the traditional way of “seeing the doctor”. Thus, in our opinion, the negative relation between access to information (HI) and paying less for medical services (PHH), is in line with the rational behavior of individuals to make their expenditures more effective, the healthcare services not being an exception.

**Figure 4:**
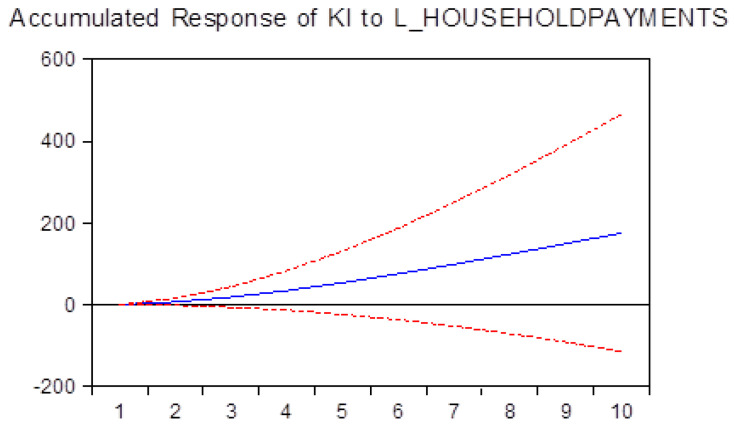
Impact of payments made by households for healthcare, expressed as euro per inhabitants on knowledge-intensive activities.

In addition, in Figure 4 we have seen that KI activities are increasing with the larger allocations of sums. Thus, our study shows that there is a rational behavior of the households that are spending larger sums in order to improve their KI activities.

## 5. Discussion

### 5.1. Research Implications

The present research demonstrates to the scientific community that Internet Usage is strongly related to knowledge intensive activities on households’ healthcare expenditures, and the longitudinal case study on European State confirms the hypothesized interrelationship. The theoretical framework modelling highlighted several identifiable benefits for households and companies for increasing the effectiveness of financial allocation on health. In addition, the recent evolution of the Covid-19 epidemic is clearly increasing the importance of Internet Health activities. Thus, the internet access will influence internet usage in a positive manner and thus, increase the efficiency of the supply chain management of the households regarding the healthcare expenditures. It makes perfect sense that this relationship works in a mutual way, both variables are influencing each other. The relationship between internet usage and internet search for seeking health related information shows a very strong correlation, an upward trend and persisted effects. Moreover, the more access to information the individuals are having, the lower the possibility of accessing healthcare services in the traditional way of “seeing the doctor”. Thus, in our opinion, the negative relation between access to information and paying less for medical services, is in line with the rational behavior of individuals to make their expenditures more effective, the healthcare services not being an exception.

Therefore, lastly, we can observe that the knowledge intensive activities are responding in a positive manner to the increase of the household payments. Therefore, we believe that there is a demand expressed in higher household payment will stimulate knowledge intensive activities.

Our study is trying to “connect the dots” between expenses and empowerment, according to WHO definition [42] and we have seen that more access to health information results in less expense for medical service which is in line with rational behavior of the households. Thus, the empowerment process is improving its effectiveness with greater access to information and also with increasing the knowledge-intensive activities. Thus, our dots that are being connected is that great internet usage together with knowledge-intensive activities and better information regarding health issues, are responsible for the household in paying less on healthcare expenditures.

### 5.2. Practical Implications

In an ever-changing economic environment due to the recent evolutions of Covid-19, or the so-called black swan phenomena, we may have to increase the importance of internet health activities and to properly regulate and finance them. This study can be useful for corporate management health system, in fact, along with the direct economic benefits for supply chain management in health system, especially for the households, “Internet Usage” may accelerate the longer-term investment in decision support system (Artificial Intelligence), offering developing countries unprecedented opportunities to enhance their predictive systems, to improve their economic policies and execution, and to improve the health care assistance. A better allocation of resources (in this case of health system) is necessary in decision-making processes regarding the investments.

### 5.3. Originality/Value

The novelty of the present study is the comparative approach made it possible to build a theoretical framework for the real evaluation of the impact of internet usage and knowledge intensive activities on households’ healthcare expenditures. Second, the research emphasizes the need to reform and reshape the studies on Supply Chain Management, convincing that it is necessary to understand that the obstacles between the Digital Divide and internet usage, i.e., must be addressed with conscious decision-making processes, strategically and resolutely, to transform points of weakness into opportunities.

### 5.4. Future Research Opportunities

A number of opportunities for future research arise from the present study. The research should stimulate researchers’ reflections on the relationship among internet usage, Supply chain management, health related activities and Digital Divide. Nevertheless, the work presented here can be considered a brief systematic study, so it is advisable to implement future research on the topic. In particular, it would be helpful to study the structure of internet health activities, their perspectives in the new COVID-19 era, in order to identify the main financial and real flows and of course the results of the internet health activities implemented by households. Such analysis may lead to the detection of the area and the implications of the internet health activities. In addition, is important to observe the manner in which the goods and services related to internet health activities are produced and marketed, and the competitive advantage for their promotion. In addition, it could be useful to replicate the methodological approach applied here in other European economic spaces, to analyse features of intra-community trade and compare them with the extra-community ones.

## 6. Conclusions

### 6.1. Search Limits

This paper is a first step to fill the gap in research on the relationship among principals’ variables. It is focused on a European panel; for this reason, it is a limited analysis which should be supplemented by future in-depth research. One of the biggest limits of our research is the time series data available on households’ preferences and activities. Information on how these preferences are shaped, modified, or altered are unavailable to academia but are accessible to big companies. Therefore, it would be necessary to conduct a systematic survey of households’ orientation in terms of internet health activities in order to validate the interrelationship, evaluated through the empirical VAR approach implemented in this paper’s case study.

Based on our results, we considered that we have proven that there is a strong relationship between household’s characteristics (analyzed in this paper) and their access to the internet. This results in the fact that when people use these internet tools to learn more about its own health issues it is correlated to making health care empowerment via the internet. As consequences, households understand their role, given the knowledge and skills acquired by their health-care provider.

### 6.2. Future Research Opportunities

A number of opportunities for future research arise from the present study. The research should stimulate researchers’ reflections on the relationship among internet usage and digital competencies for health decision making processes and also for observing the results. Nevertheless, the work presented here can be considered a brief systematic study, so it is advisable to implement future research on the topic. In particular, it would be helpful to study the structure of the main goods that are being marketed and traded using the Internet, in order to identify the main financial and real flows indicators. In addition, it could be useful to replicate the methodological approach applied here in other European economic spaces, to analyze features of intra-community health scenarios and compare them with the extra-community ones. In the future we should consider an analysis on the sustainability of healthcare behavior of households to analyze community and cultural differences about patient participation.

## Figures and Tables

**Table 1:** VAR stability condition.

Root	Modulus
0.985	0.985
0.920	0.920
0.908	0.908
0.810 − 0.012i	0.810
0.810 + 0.012i	0.810
−0.211	0.211
−0.129	0.129
0.101	0.101
−0.031 − 0.082i	0.088
−0.031 + 0.082i	0.088

No root lies outside the unit circle. VAR satisfies the stability condition.

**Table 2:** VAR Lag order selection.

Lag	LogL	LR	FPE	AIC	SC	HQ
0	−1026.059	NA	2.64 × 10^10^	38.18	38.37	38.25
1	−688.29	600.46	247,400.2 *	26.60	27.70 *	27.02 *
2	−671.48	26.76	343,363.9	26.90	28.93	27.68
3	−654.45	23.97	491,056.5	27.20	30.14	28.33
4	−621.81	39.89 *	419,032.8	26.91	30.78	28.41
5	−607.39	14.95	771,252.6	27.31	32.09	29.15
6	−588.31	16.25	1,377,482.3	27.53	33.23	29.73
7	−555.87	21.62	1,878,280.4	27.25	33.88	29.81
8	−505.06	24.46	1,907,265.6	26.29 *	33.84	29.21

* indicates lag order selected by the criterion; LR: sequential modified LR test statistic (each test at 5% level); FPE: Final prediction error; AIC: Akaike information criterion; SC: Schwarz information criterion; HQ: Hannan-Quinn information criterion.

**Table 3:** Autocorrelation Portmanteau test.

Lags	Q-Stat	Prob.	Adj Q-Stat	Prob.	df
1	17.12	NA *	17.19	NA *	NA *
2	36.53	0.06	36.76	0.06	25

* The test is valid only for lags larger than the VAR lag order; df is degrees of freedom for (approximate) chi-square distribution.

**Table 4:** VAR Residual Heteroskedasticity test.

Joint Test		
Chi-sq	df	Prob.
211.9654	150	0.0007
